# Supplementary material for: Deficiencies in the Fanconi anemia or homologous recombination pathway enhance the antitumor effects of the hypoxia-activated prodrug CP-506
Source: Mol Ther Oncol. 2026 Feb 24;34(1):201161. doi: 10.1016/j.omton.2026.201161 (PMC12995515; doi:10.1016/j.omton.2026.201161)
Supplement: Document S1. Figures S1–S10, Tables S1–S4, and supplemental materials and methods [file mmc1.pdf]

OMTON, Volume *34*

Supplemental information

Deficiencies in the Fanconi anemia or homologous recombination pathway enhance the antitumor effects of the hypoxia-activated prodrug CP-506

Lesley Schuitmaker, Alexander M.A. van der Wiel, Natasja G. Lieuwes, Rianne Biemans, Nikki A.M. Mutsters, Jennifer Jung, Victoria Claudino Bastos, Èlia Prades Sagarra, Sheng Kuang, Jeremy Setton, Sabine A.S. Langie, Kim R. Kampen, Jan Theys, Ala Yaromina, Ludwig J. Dubois, and Philippe Lambin

Supplemental Figures

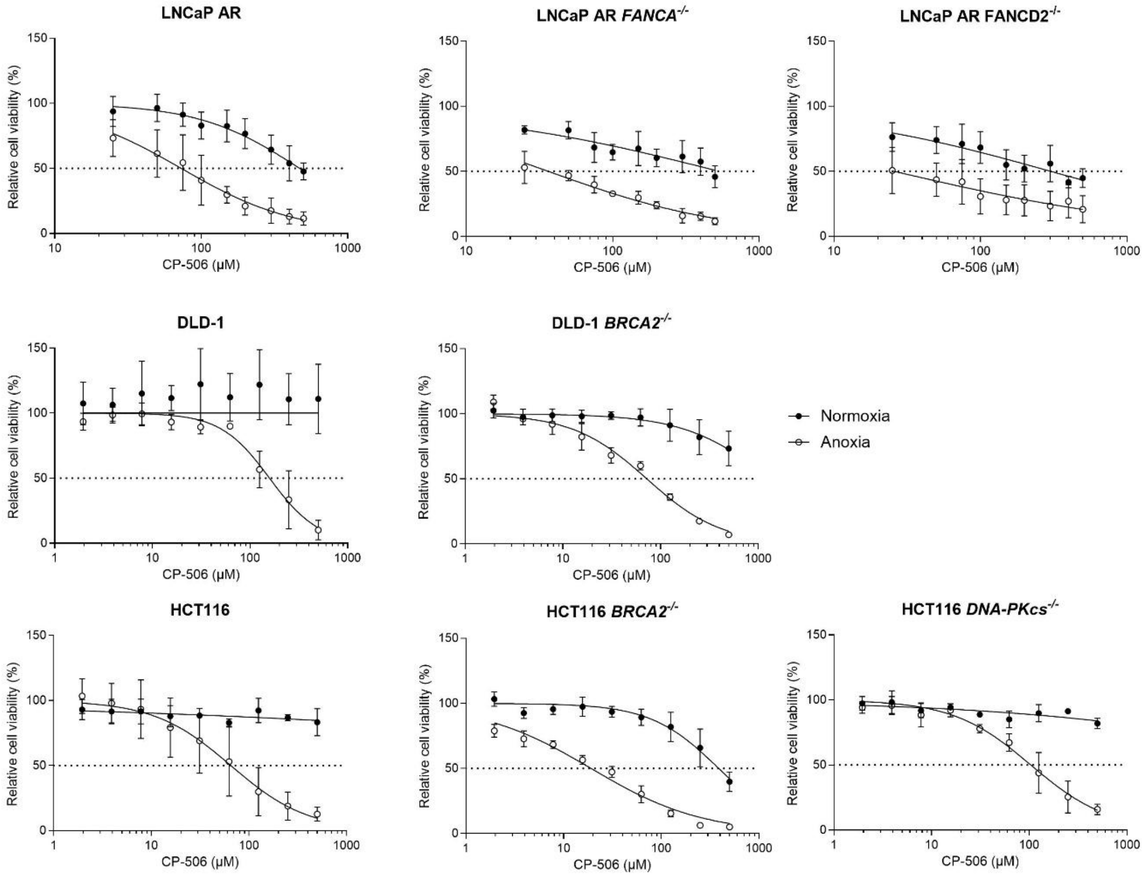

Figure S1. Cell viability dose-response curves upon CP-506 exposure in isogenic cancer cell lines proficient or deficient in the FA *(FANCA*$^{-/-}$ or *FANCD2*$^{-/-}$*),* HR *(BRCA2*$^{-/-}$*),* or NHEJ *(DNA-PKcs*$^{-/-}$*)* DNA repair pathways. Isogenic cancer cells were exposed to increasing CP-506 concentrations under normoxic (closed circles) and anoxic (open circles) conditions after which cell viability was assessed and IC_{50} values were determined. Data represent mean ± SEM of 3 biological repeats.

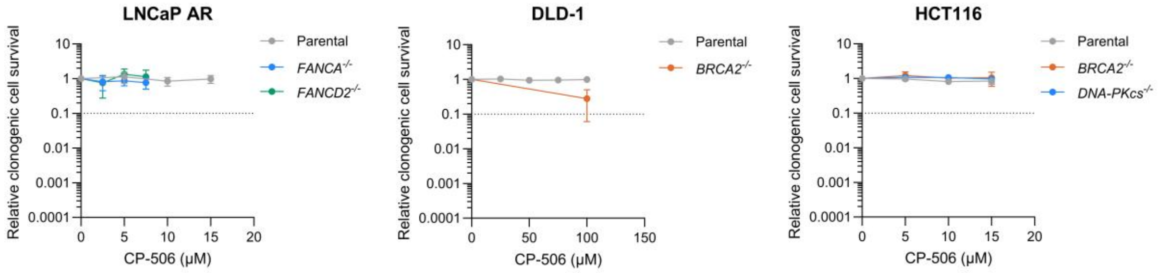

Figure S2. Clonogenic cell survival after 4 hours of exposure to CP-506 of isogenic cancer cell lines proficient or deficient in DNA repair pathways upon normoxic conditions. Data represent mean ± SD of ≥ 3 biological repeats.

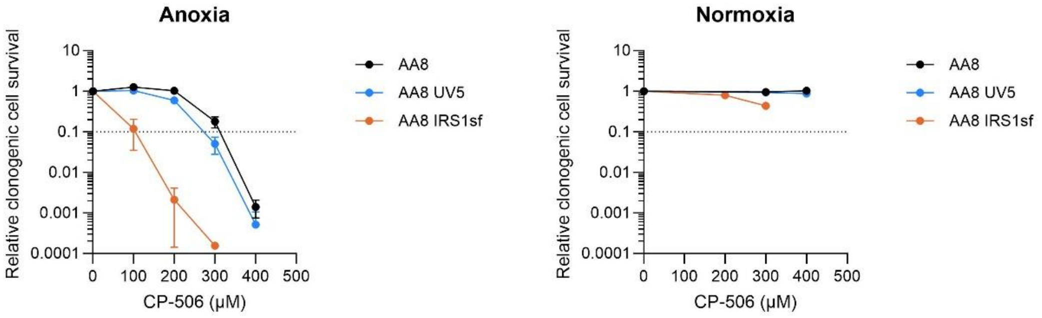

Figure S3. Clonogenic cell survival of AA8 cells after 4 hours of anoxic or normoxic exposure to CP-506. Data represent mean ± SD of 3 biological repeats.

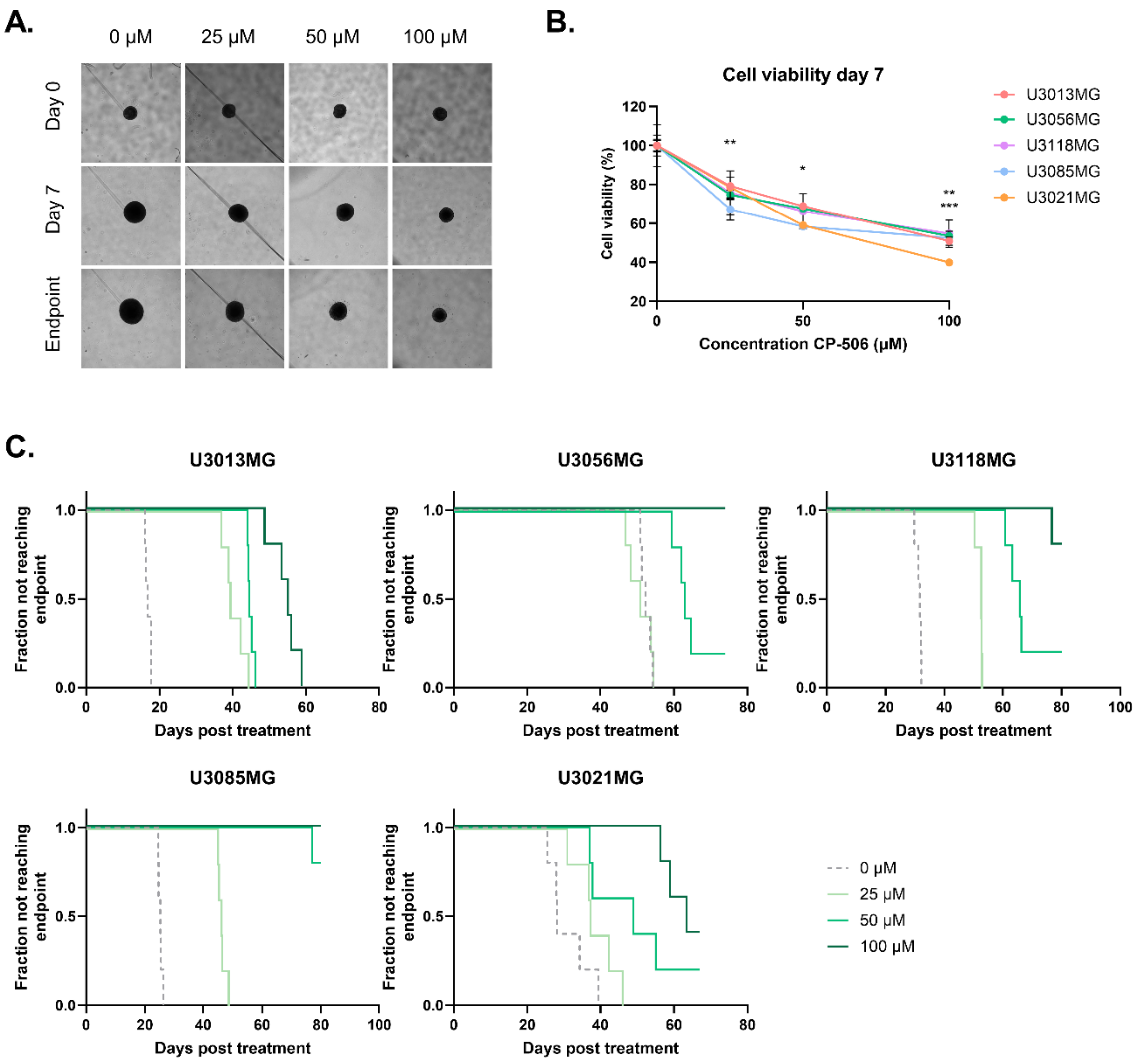

Figure S4. CP-506 treatment response in glioblastoma spheroids. (A) Representative images of U3013MG spheroids at the day of CP-506 treatment (day 0), the day of cell viability assessment (day 7), and at the experimental endpoint (defined as the time at which vehicle-treated spheroids reached 5xSV). (B) Cell viability of GBM spheroids as assessed with alamarBlue™ reagent on day 7 post treatment following exposure to increasing CP-506 concentrations (25 µM, 50 µM, and 100 µM). (C) Spheroid growth was monitored and survival, defined as the fraction of spheroids not reaching endpoint, was assessed. n = 5 spheroids per condition. Data are presented as mean ± SD. *: $P < 0.05$, **: $P < 0.01$, ***: $P < 0.001$.

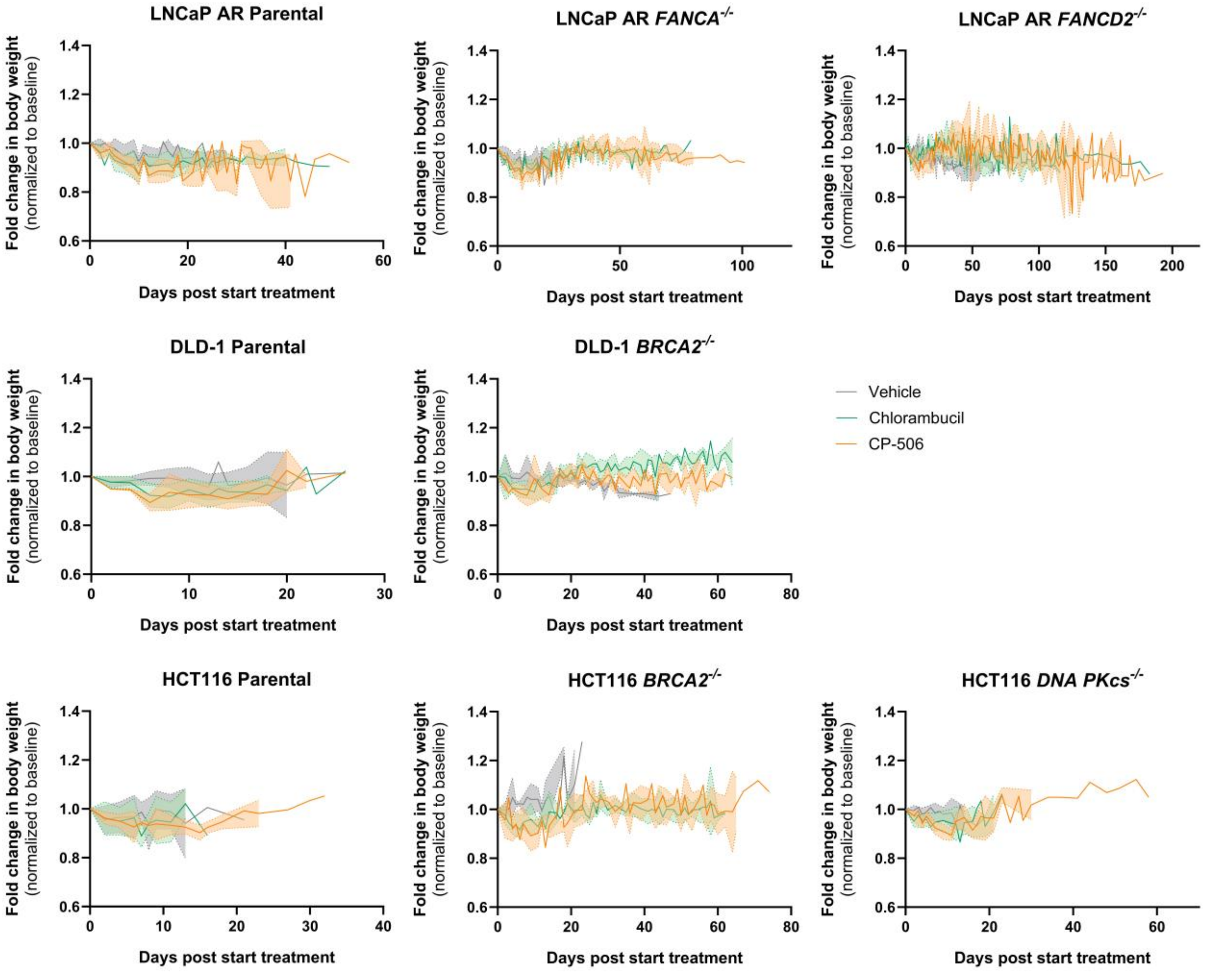

Figure S5. Effect of vehicle, chlorambucil, or CP-506 treatment on the body weight of mice bearing isogenic tumor xenografts. Body weight changes over time upon vehicle (WFI), chlorambucil, or CP-506 treatment, normalized to body weight at the first day of treatment. Data are shown as mean ± SD (n = 8-10 animals per group).

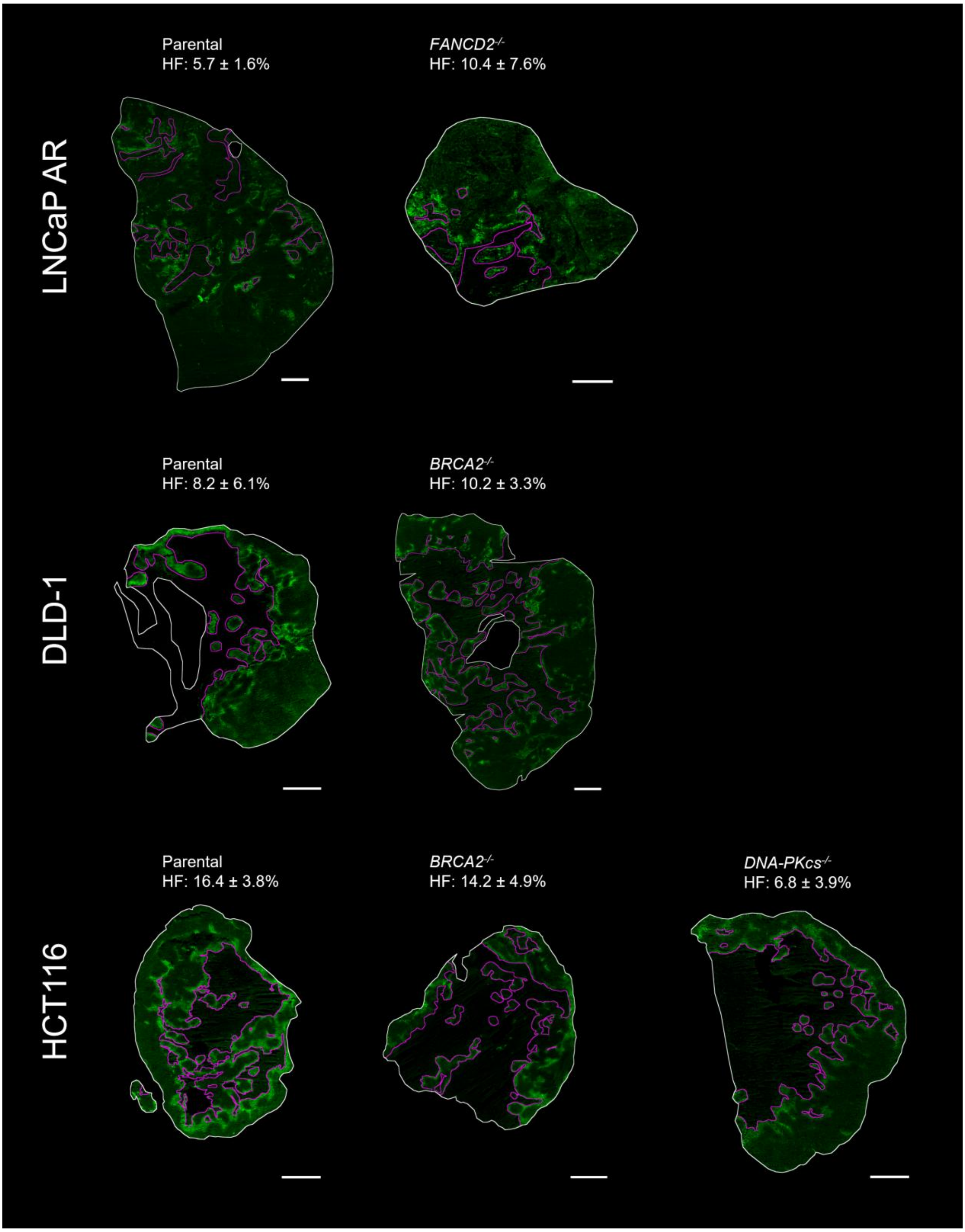

Figure S6. Pimonidazole hypoxic fraction (HF) in isogenic xenografts 6 hours post vehicle treatment. Representative images showing total tumor area (white) and necrotic tumor area (magenta); background outside the total tumor area was cleared for visualization. Data are reported as mean ± SD (n = 4-6 animals per group). Scale bar represents 1 mm.

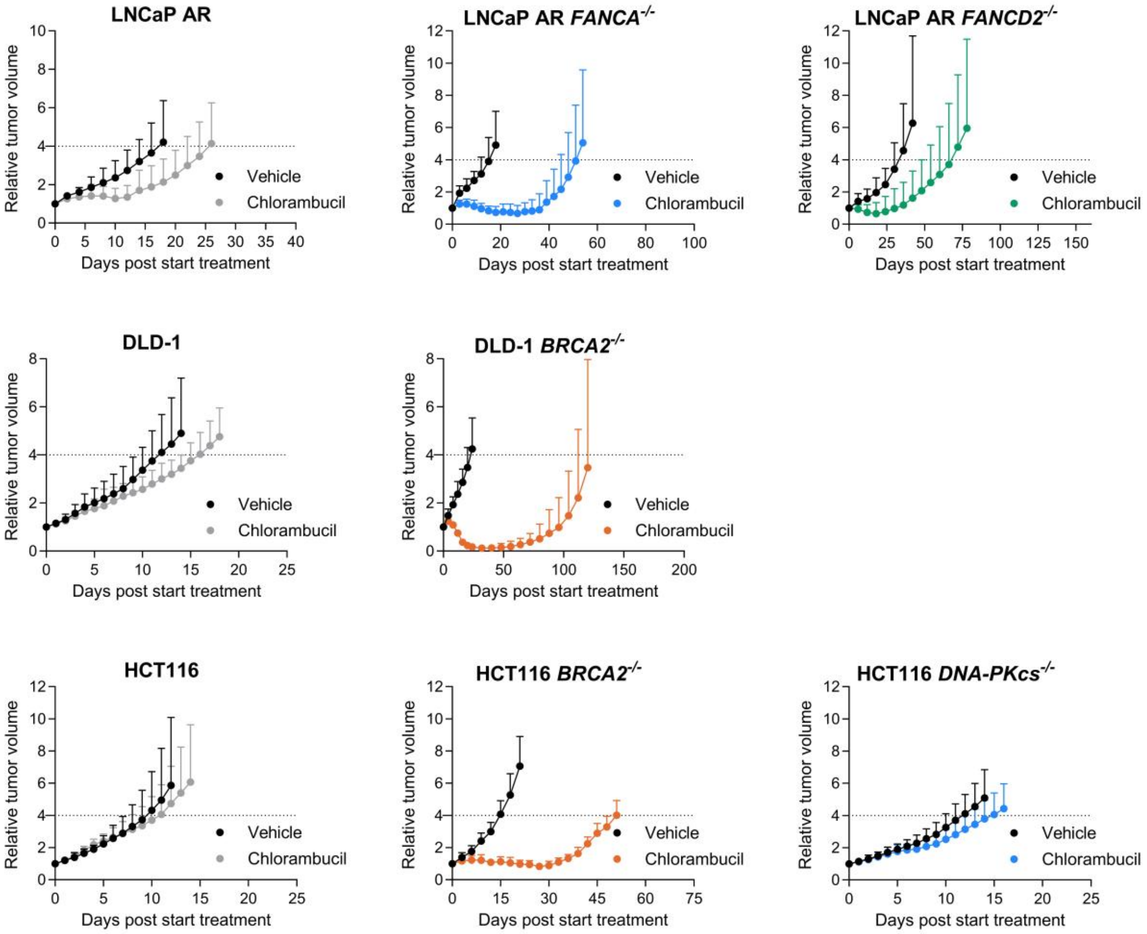

Figure S7. Antitumor effects of vehicle (WFI) or chlorambucil treatment (3 mg/kg, QD5, IP) in mice bearing isogenic tumor xenografts, proficient or deficient in FA (*FANCA*$^{-/-}$ or *FANCD2*$^{-/-}$), HR (*BRCA2*$^{-/-}$), or NHEJ (*DNA-PKcs*$^{-/-}$). Data are presented as mean ± SD (n = 8-10 animals per group).

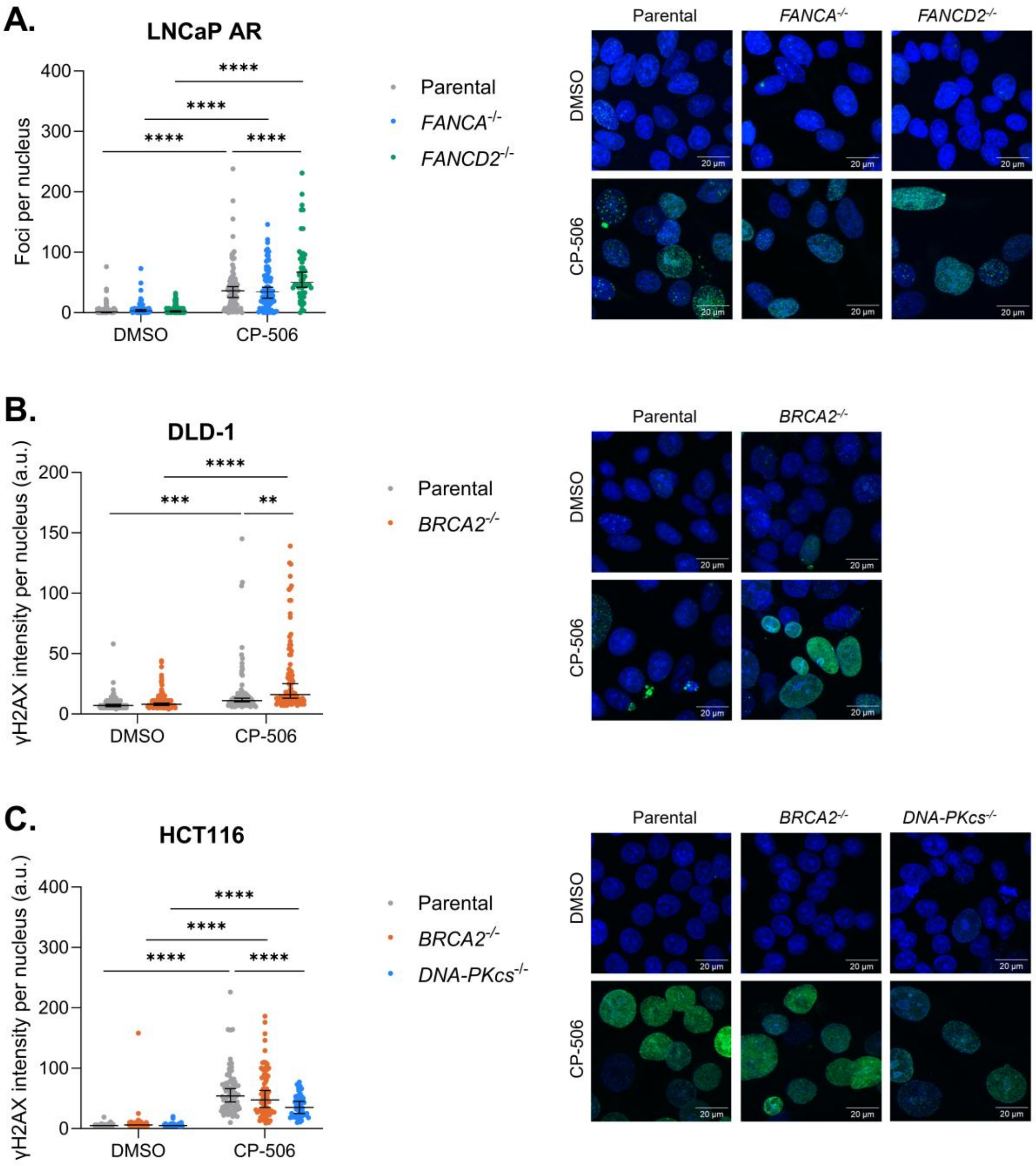

Figure S8. CP-506 induced persistent DNA damage in FA- and HR-deficient isogenic cancer cells under anoxic conditions as determined by immunofluorescence 72 hours post start of treatment. Foci count per nucleus for LNCaP AR isogenic cancer cells (A) and quantification of γH2AX immunofluorescence intensity per nucleus for DLD-1 (B) and HCT116 (C) isogenic cancer cells with representative γH2AX immunofluorescence images. Blue: Hoechst; green: γH2AX. n ≥ 57 cells per condition and data are presented as median (IQR). *: $P < 0.05$, **: $P < 0.01$, ***: $P < 0.001$, ****: $P < 0.0001$.

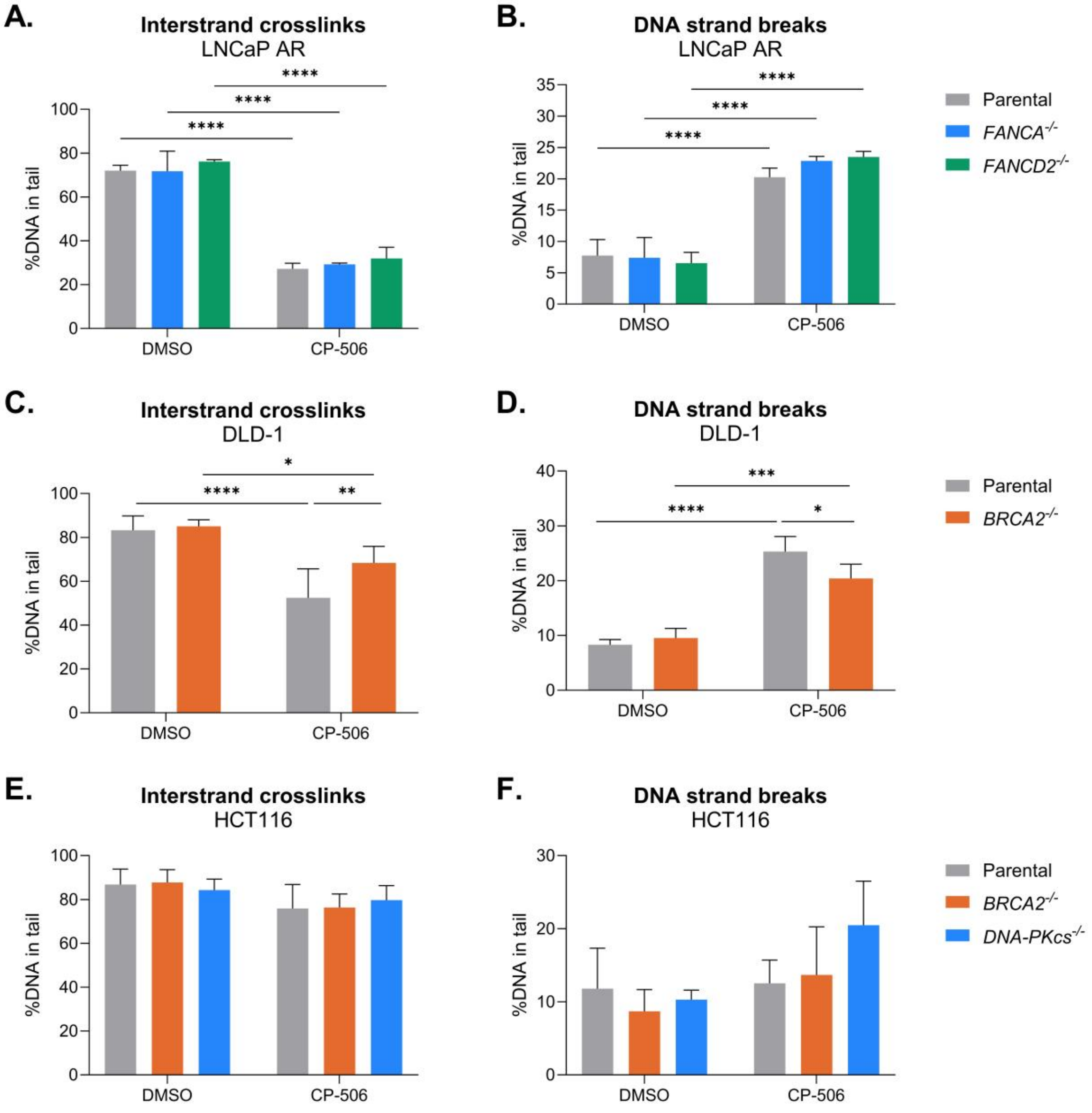

Figure S9. Anoxic exposure to CP-506 induced ICLs and DNA strand breaks in isogenic cancer cells 72 hours post treatment. Comet assay analysis of isogenic LNCaP AR, DLD-1, and HCT116 cells exposed to DMSO or CP-506 under anoxic conditions. Cells were either assessed for interstrand crosslinks (ICLs; A, C, E) or DNA strand breaks (SSBs and DSBs; B, D, F). Medians from two biological repeats with one or more technical repeats were averaged ± SD. *: $P < 0.05$, **: $P < 0.01$, ***: $P < 0.001$, ****: $P < 0.0001$.

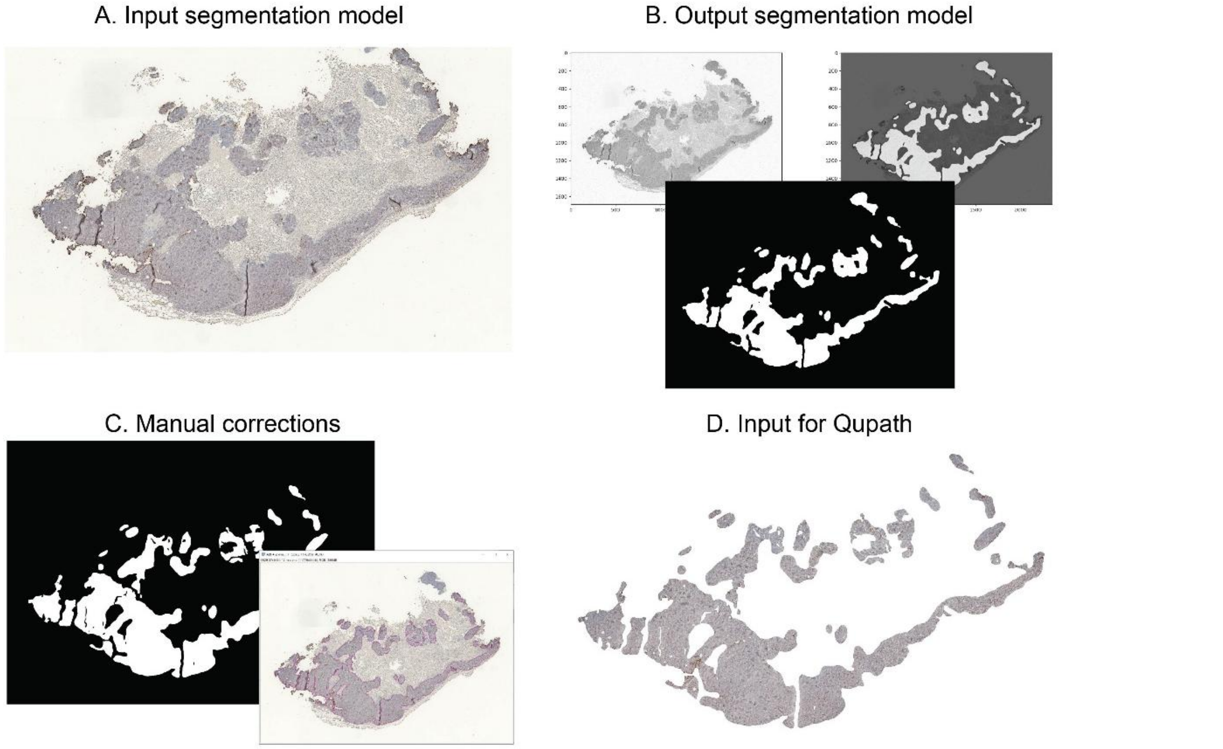

Figure S10. Workflow for the segmentation of vital masks of isogenic tumor sections for the analysis of γH2AX immunohistochemistry staining. A deep-learning DynUNet model was generated to segment the vital tumor areas from raw RGB images. After performing manual corrections of the segmentations using ImageJ version 1.54f, the corrected vital mask was overlaid with the raw RGB image, which generates an input image in Qupath version 0.4.3. for immunohistochemistry analysis of γH2AX (% positive cells).

Supplemental Tables

Table S1. Glioblastoma spheroid growth delay and spheroid growth inhibition upon CP-506 treatment (25 µM, 50 µM, and 100 µM) normalized to vehicle-treated spheroids. Data are presented as mean ± SD, n = 5 spheroids per condition.

Cell line	Sex (age)	GBM subtype	DNA repair score			SGD (days)			SGI (%)		
			FA	HR	Combined	25 µM	50 µM	100 µM	25 µM	50 µM	100 µM
U3013MG	F (78)	Proneural	0.80	0.85	0.82	23.5±2.9	28.0±0.9	37.5±3.7	72.7±6.1	91.5±2.0	98.7±0.8
U3056MG	F (68)	Classical	0.45	0.71	0.57	-1.7±3.3	9.8±2.2[A]	NA	-29.7±50.1	72.0±17.5	99.4±0.2
U3118MG	M (57)	Proneural	0.06	0.19	0.13	21.0±1.1	32.7±2.6[A]	45.4[C]	81.7±3.0	96.5±1.7	98.8±0.6
U3085MG	M (68)	Neural	-0.64	-0.10	-0.37	21.1±1.4	51.8[C]	NA	82.2±1.7	91.8±3.7	92.4±0.2
U3021MG	M (50)	Neural	-0.24	-0.62	-0.43	3.2±5.8	9.3±8.7[A]	24.1±3.6[B]	6.6±15.3	31.1±20.1	82.2±7.5

GBM: glioblastoma; FA: Fanconi anemia pathway; HR: homologous recombination; SGD: spheroid growth delay; SGI: spheroid growth inhibition

[A] n = 4 spheroids reaching endpoint

[B] n = 3 spheroids reaching endpoint

[C] n = 1 spheroid reaching endpoint

NA: not available, as no spheroids reached endpoint

Table S2. Read-out parameters monotherapeutic efficacy CP-506 in isogenic xenograft models.

Cell line	Cancer type	DNA repair pathway	Mouse strain	T4xSV – vehicle	T4xSV – CP-506	ER	TGI	P-value T4xSV vehicle vs CP-506	P-value ER vs WT	P-value TGI vehicle vs CP-506
LNCaP AR	Prostate	Parental	NOD.Cg-Prkdc^{SCID} Il2rg^{tm1Wjl}/SzJ	19.2 ± 5.8	29.4 ± 9.0	1.5 ± 0.5	52.3 ± 56.4%	0.29	-	< 0.05
LNCaP AR *FANCA*$^{-/-}$	Prostate	FA		16.6 ± 4.2	66.7 ± 18.3	4.0 ± 1.1	98.6 ± 1.5%	< 0.0001	< 0.0001	< 0.0001
LNCaP AR *FANCD2*$^{-/-}$	Prostate	FA		40.6 ± 13.4	139.0 ± 30.5	3.4 ± 0.8	97.7 ± 3.2%	< 0.0001	< 0.0001	< 0.0001
DLD-1	Colorectal	Parental	CAnN.Cg-Foxn1^{nu}/Crl	13.9 ± 4.6	18.3 ± 2.8	1.3 ± 0.2	37.9 ± 10.3%	0.49	-	< 0.01
DLD-1 *BRCA2*$^{-/-}$	Colorectal	HR		25.5 ± 7.8	73.5 ± 17.9	2.9 ± 0.7	77.8 ± 11.0%	< 0.0001	< 0.0001	<0.0001
HCT116	Colorectal	Parental	Crl:NU-Foxn1^{nu}	10.2 ± 3.8	17.0 ± 5.7	1.7 ± 0.6	59.5 ± 12.9%	< 0.01	-	< 0.01
HCT116 *BRCA2*$^{-/-}$	Colorectal	HR		15.3 ± 2.3	61.4 ± 9.2	4.0 ± 0.6	95.7 ± 3.2%	< 0.0001	< 0.0001	< 0.0001
HCT116 *DNA-PKcs*$^{-/-}$	Colorectal	NHEJ		12.6 ± 2.7	17.5 ± 3.6	1.4 ± 0.3	33.2 ± 17.3%	< 0.05	0.18	0.15

T4xSV: time to reach 4 times start volume; ER: enhancement ratio; TGI: tumor growth inhibition at the day which respective vehicle-treated tumors reached four times start volume (4xSV).

Table S3.
An overview of all isogenic cell lines, their tissue of origin, genetic mutation and corresponding DNA repair pathway affected, provider, and culture medium used.

Cell line	Cancer type	DNA repair pathway	Provider	Culture medium
LNCaP AR	Prostate (androgen resistant)	Parental	MSKCC	DMEM, supplemented with 8-10% FBS
LNCaP AR *FANCA*$^{-/-}$	Prostate (androgen resistant)	FA	MSKCC	DMEM, supplemented with 10% FBS
LNCaP AR *FANCD2*$^{-/-}$	Prostate (androgen resistant)	FA	MSKCC	DMEM, supplemented with 10% FBS
LNCaP AR *ATM*$^{-/-}$	Prostate (androgen resistant)	DSB	MSKCC	DMEM, supplemented with 8% FBS
LNCaP AR *CHEK2*$^{-/-}$	Prostate (androgen resistant)	DSB	MSKCC	DMEM, supplemented with 8% FBS
DLD-1	Colorectal	Parental	Horizon Discovery	McCoy's 5A, supplemented with 10% FBS
DLD-1 *BRCA2*$^{-/-}$	Colorectal	HR	Horizon Discovery	McCoy's 5A, supplemented with 10% FBS
HCT116	Colorectal	Parental	Horizon Discovery	DMEM, supplemented with 10% FBS
HCT116 *BRCA2*$^{-/-}$	Colorectal	HR	Ximbio	DMEM, supplemented with 10% FBS
HCT116 *DNA-PKcs*$^{-/-}$	Colorectal	NHEJ	Horizon Discovery	DMEM, supplemented with 10% FBS
AA8	Chinese hamster ovary	Parental	UZH	Ham’s F-10 Nutrient Mix, supplemented with 8% FBS
AA8 UV5 (*XPD*$^{-/-}$)	Chinese hamster ovary	NER	UZH	Ham’s F-10 Nutrient Mix, supplemented with 8% FBS
AA8 IRS1sf (*XRCC3*$^{-/-}$)	Chinese hamster ovary	HR	UZH	Ham’s F-10 Nutrient Mix, supplemented with 8% FBS

FA: Fanconi anemia pathway; DSB: double strand break; HR: homologous recombination; NHEJ: non-homologous end joining; NER; nucleotide excision repair; FANCA: FA complementation group A; FANCD2: FA complementation group D2; BRCA2: breast cancer type 2 susceptibility protein; DNA-PKcs: DNA-dependent protein kinase catalytic subunit; UV5: ultraviolet sensitive mutant 5; XPD: xeroderma pigmentosum D orthologue; IRS1sf: ionizing irradiation sensitive close 1 sf; XRCC3: X-ray repair cross-complementing protein 3; MSKK: Memorial Sloan Kettering Cancer Center; UZH: University Hospital Zurich; DMEM: Dulbecco's Modified Eagle Medium; FBS: fetal bovine serum.

Table S4. An overview of the optimized settings per isogenic tumor model for positive cell detection in QuPath version 0.4.3

Parameter	LNCaP AR	DLD-1	HCT116
**Setup parameters**			
Detection image	Optical density sum	Optical density sum	Optical density sum
Requested pixel size	0.5 µm	0.5 µm	0.5 µm
**Nucleus parameters**			
Background radius	85.0 µm	8.0 µm	50.0 µm
Use opening by reconstruction	TRUE	FALSE	FALSE
Medium filter radius	0.0 µm	0.0 µm	0.0 µm
Sigma	2.0 µm	1.4 µm	1.3 µm
Minimum area	20.0 μm^2	10.0 μm^2	13.0 μm^2
Maximum area	400.0 μm^2	400.0 μm^2	400.0 μm^2
**Intensity parameters**			
Threshold	0.26	0.16	0.16
Max background intensity	10.0	2.0	2.0
Split by shape	TRUE	TRUE	TRUE
Exclude DAB (membrane staining)	FALSE	FALSE	FALSE
**Cell parameters**			
Cell expansion	1.0 µm	1.0 µm	1.0 µm
Include cell nulcues	TRUE	TRUE	TRUE
**General parameters**			
Smooth boundaries	TRUE	TRUE	TRUE

Make measurements	TRUE	TRUE	TRUE
**Intensity threshold parameters**			
Score compartment	Nucleus: DAB OD mean	Nucleus: DAB OD mean	Nucleus: DAB OD mean
Threshold 1+	0.26	0.30	0.20
Threshold 2+	(0.4)	(0.4)	(0.4)
Threshold 3+	(0.6)	(0.6)	(0.6)
Single threshold	TRUE	TRUE	TRUE

Script for the analysis of γH2AX immunohistochemistry staining in LNCaP AR isogenic tumors:

```
setImageType('BRIGHTFIELD_H_DAB');
setColorDeconvolutionStains('{"Name" : "H-DAB estimated", "Stain 1" : "Hematoxylin", "Values 1" : "0.62644 0.62296 0.46851", "Stain 2" : "DAB", "Values 2" : "0.39554 0.53531 0.74632", "Background" : " 238 239 233"}');
runPlugin('qupath.imagej.detect.tissue.SimpleTissueDetection2', '{"threshold":212,"requestedPixelSizeMicrons":2.0,"minAreaMicrons":10000.0,"maxHoleAreaMicrons":1000.0,"darkBackground":false,"smoothImage":true,"medianCleanup":true,"dilateBoundaries":false,"smoothCoordinates":true,"excludeOnBoundary":false,"singleAnnotation":true}')
selectAnnotations();
runPlugin('qupath.imagej.detect.cells.PositiveCellDetection', '{"detectionImageBrightfield":"Optical density sum","requestedPixelSizeMicrons":0.5,"backgroundRadiusMicrons":85.0,"backgroundByReconstruction":true,"medianRadiusMicrons":0.0,"sigmaMicrons":2.0,"minAreaMicrons":20.0,"maxAreaMicrons":400.0,"threshold":0.26,"maxBackground":10.0,"watershedPostProcess":true,"excludeDAB":false,"cellExpansionMicrons":1.0,"includeNuclei":true,"smoothBoundaries":true,"makeMeasurements":true,"thresholdCompartment":"Nucleus: DAB OD mean","thresholdPositive1":0.26,"thresholdPositive2":0.4,"thresholdPositive3":0.6000000000000001,"singleThreshold":true}')
```

Script for the analysis of γH2AX immunohistochemistry staining in DLD-1 isogenic tumors:

```
setImageType('BRIGHTFIELD_H_DAB');
setColorDeconvolutionStains('{"Name" : "H-DAB estimated", "Stain 1" : "Hematoxylin", "Values 1" : "0.63305 0.61773 0.46654", "Stain 2" : "DAB", "Values 2" : "0.36417 0.57223 0.7348", "Background" : " 242 244 239"}');
runPlugin('qupath.imagej.detect.tissue.SimpleTissueDetection2', '{"threshold":212,"requestedPixelSizeMicrons":2.0,"minAreaMicrons":10000.0,"maxHoleAreaMicrons":1000.0,"darkBackground":false,"smoothImage":true,"medianCleanup":true,"dilateBoundaries":false,"smoothCoordinates":true,"excludeOnBoundary":false,"singleAnnotation":true}')
selectAnnotations();
```

```
runPlugin('qupath.imagej.detect.cells.PositiveCellDetection', '{"detectionImageBrightfield":"Optical density sum","requestedPixelSizeMicrons":0.5,"backgroundRadiusMicrons":8.0,"backgroundByReconstruction":false,"medianRadiusMicrons":0.0,"sigmaMicrons":1.4,"minAreaMicrons":10.0,"maxAreaMicrons":400.0,"threshold":0.16,"maxBackground":2.0,"watershedPostProcess":true,"excludeDAB":false,"cellExpansionMicrons":1.0,"includeNuclei":true,"smoothBoundaries":true,"makeMeasurements":true,"thresholdCompartment":"Nucleus: DAB OD mean","thresholdPositive1":0.3,"thresholdPositive2":0.4,"thresholdPositive3":0.6000000000000001,"singleThreshold":true}')
runObjectClassifier("connective tissue DLD-1");
```

Script for the analysis of γH2AX immunohistochemistry staining in HCT116 isogenic tumors:

```
setImageType('BRIGHTFIELD_H_DAB');
setColorDeconvolutionStains('{"Name" : "H-DAB estimated", "Stain 1" : "Hematoxylin", "Values 1" : "0.62714 0.62104 0.47012", "Stain 2" : "DAB", "Values 2" : "0.45303 0.56698 0.68796", "Background" : " 244 243 236"}');
runPlugin('qupath.imagej.detect.tissue.SimpleTissueDetection2', '{"threshold":212,"requestedPixelSizeMicrons":2.0,"minAreaMicrons":10000.0,"maxHoleAreaMicrons":1000.0,"darkBackground":false,"smoothImage":true,"medianCleanup":true,"dilateBoundaries":false,"smoothCoordinates":true,"excludeOnBoundary":false,"singleAnnotation":true}')
selectAnnotations();
runPlugin('qupath.imagej.detect.cells.PositiveCellDetection', '{"detectionImageBrightfield":"Optical density sum","requestedPixelSizeMicrons":0.5,"backgroundRadiusMicrons":50.0,"backgroundByReconstruction":false,"medianRadiusMicrons":0.0,"sigmaMicrons":1.3,"minAreaMicrons":13.0,"maxAreaMicrons":400.0,"threshold":0.16,"maxBackground":2.0,"watershedPostProcess":true,"excludeDAB":false,"cellExpansionMicrons":1.0,"includeNuclei":true,"smoothBoundaries":true,"makeMeasurements":true,"thresholdCompartment":"Nucleus: DAB OD mean","thresholdPositive1":0.2,"thresholdPositive2":0.4,"thresholdPositive3":0.6000000000000001,"singleThreshold":true}')
```

Supplemental Materials and Methods

Clonogenic cell survival assay

AA8 cells were seeded in 60-mm glass dishes in optimized cell densities. After 4 hours of incubation with CP-506-containing pre-incubated anoxic culture medium, cells were transferred to normoxic conditions, washed, harvested, and seeded as single cells to assess clonogenic cell survival after ~10 days. Colonies (> 50 cells) were manually counted to determine plating efficiency, after which survival fractions were calculated and analyzed with Graphpad Prism 10.1.2 software (GraphPad Software, Inc.). Tissue of origin, genetic mutation and corresponding DNA repair pathway affected, provider, and culture medium are reported in Table S3. AA8 cells were a kind gift from Prof. Pruschy.

Glioblastoma spheroid assay

Patient-derived glioblastoma cells (U3013MG, U3021MG, U3056MG, U3085MG, and U3118MG) were acquired from the Human Glioma Cell Cultures (HGCC) resource (www.hgcc.se) at the Dept. of Immunology, Genetics and Pathology, Uppsala University, Uppsala, Sweden and cultured as described previously [1]. In short, cell cultures were maintained in Neurobasal and advanced DMEM/F12 media (1:1) supplemented with N-2 and B27 supplements (Gibco), GlutaMAX (Gibco), human recombinant EGF and FGFb (10 ng/ml; PeproTech), and Primocin (25 µg/ml; Invivogen). For spheroid formation, 2000 cells/well were seeded in 96-wells ULA plates. Monitoring spheroid growth and determining spheroid volume were performed as described in the main manuscript. On day 14, spheroids were treated with CP-506 for 24 hours, after which spheroids were washed, and culture medium was refreshed twice a week. Treatment response was quantified as spheroid growth delay (SGD), i.e. the difference in time to reach endpoint in CP-506-treated spheroids compared to vehicle-treated spheroids, and spheroid growth inhibition (SGI) at endpoint.

On day 7 post treatment, cell viability was measured using alamarBlue™ reagent upon 24 hour incubation for U3013MG, U3021MG, and U3056MG or 48 hour incubation for U3085MG and U3118MG spheroids. Cell viability was normalized to vehicle-control treated spheroids. To test statistical significance, a two-way ANOVA was performed.

Calculation of DNA repair proficiency score

Gene expression data were extracted from the HGCC database, and an FA, HR, and combined DNA repair proficiency score was calculated. Specifically, FA (FANCA, FANCB, FANCC, FANCD2, FANCE, FANCF, FANCG, FANCI, FANCL, FANCM, UBE2T, SLX4, REV1, FAN1, USP1) and HR (BRCA1, BRCA2, PALB2, RAD51, RAD51C, XRCC2, XRCC3, BARD1, BRIP1, MRE11A, RAD50, NBN, RBBP8, EXO1, BLM, GEN1, MUS81, EME1, WRN, RECQL, RECQL4) genes were extracted and median z-scores were calculated for the FA and HR repair proficiency scores, respectively. The combined DNA repair proficiency score was calculated as followed: $\frac{(median\ FA + median\ HR)}{2}$.

Assessment of tumor hypoxia by pimonidazole immunofluorescence

To assess the hypoxic fraction (HF), tumor sections were stained for the hypoxia marker pimonidazole (NPI, Inc) as previously described [2,3]. In short, frozen tumor sections (7 µm) were fixed in cold acetone and blocked with 5% (v/v) normal goat serum in antibody diluent. Overnight incubation (4 °C) with rabbit anti-pimonidazole antibody (1:250 in antibody diluent; NPI, Inc.) was followed by TBS-Tw washes and a one-hour incubation with goat anti-rabbit IgG Alexa Fluor 488 (1:500 in antibody diluent; Invitrogen) at RT. Sections were incubated with DAPI for 10 minutes and mounted in fluorescent mounting medium (DakoCytomation). Images were acquired with an Olympus BX51WI microscope

equipped with a 10x objective, Hamamatsu EM-CCD C9100 digital camera, and a Ludl Mac 2000 motorized stage. For quantitative image analysis, viable tumor tissue was delineated manually based on DAPI with ImageJ (N.G.L. and R.B.) blinded to the subject coding. Thereafter, thresholds were set manually to distinguish pimonidazole fluorescence from background and HF was calculated as the ratio of the pimonidazole positive area to the viable tumor area.

Vital tumor segmentation using Deep-learning DynUNet models

A DynUNet [4] architecture (implemented using Python 3.11.5 and Monai 1.3.0) was employed to delineate vital tumor areas, excluding necrotic tumor areas, connective tissues, and processing or staining artefacts, within the scanned slides. For the input to the DynUNet architecture, two observers (N.G.L. and L.S.) manually annotated a randomly selected subset of 30 scanned slides in RGB format using ImageJ software 1.54f.

The dataset was stratified into a training set consisting of 22 images and a validation set comprising 8 images ensuring equal contribution of the different isogenic tumor models. To reduce the computational complexity, the RGB images were first down sampled by a factor of 5 using bilinear interpolation. During training, 32 patches (512 x 512 pixels) were randomly extracted from each image and data augmentation techniques, including random adjustments in contrast, intensity, rotation, and flip, were applied.

For training the DynUNet, a deep supervision mechanism was incorporated, ensuring robust learning at multiple network depths. A combined Dice and cross-entropy loss was employed to optimize segmentation performance. The Adam optimizer was used for 1000 epochs with an initial learning rate of 0.002, following a polynomial decay schedule. The model with the best validation performance was retrained for final interference.

During inference, a sliding window approach with a stride of 384 was used to ensure comprehensive coverage and precision in the prediction of vital areas. The outputs from overlapping patches were integrated using Gaussian fusion. The final segmentation mask was obtained by applying a threshold of 0.5 to the prediction probabilities, after which the mask was up sampled back to match the original image resolution using nearest interpolation.

References

1. Xie, Y., Bergstrom, T., Jiang, Y., Johansson, P., Marinescu, V.D., Lindberg, N., Segerman, A., Wicher, G., Niklasson, M., Baskaran, S., et al. (2015). The Human Glioblastoma Cell Culture Resource: Validated Cell Models Representing All Molecular Subtypes. EBioMedicine *2*, 1351-1363. 10.1016/j.ebiom.2015.08.026.

2. Yaromina, A., Koi, L., Schuitmaker, L., van der Wiel, A.M.A., Dubois, L.J., Krause, M., and Lambin, P. (2023). Overcoming radioresistance with the hypoxia-activated prodrug CP-506: A pre-clinical study of local tumour control probability. Radiother Oncol *186*, 109738. 10.1016/j.radonc.2023.109738.

3. van der Wiel, A.M.A., Jackson-Patel, V., Niemans, R., Yaromina, A., Liu, E., Marcus, D., Mowday, A.M., Lieuwes, N.G., Biemans, R., Lin, X., et al. (2021). Selectively Targeting Tumor Hypoxia With the Hypoxia-Activated Prodrug CP-506. Molecular Cancer Therapeutics. 10.1158/1535-7163.Mct-21-0406.

4. Cardoso, M.J., Li, W., Brown, R., Ma, N., Kerfoot, E., Wang, Y., Murrey, B., Myronenko, A., Zhao, C., and Yang, D. (2022). Monai: An open-source framework for deep learning in healthcare. arXiv preprint arXiv:2211.02701.
